# Cholinesterase assay by an efficient fixed time endpoint method

**DOI:** 10.1016/j.mex.2014.10.010

**Published:** 2014-11-04

**Authors:** Mónica Benabent, Eugenio Vilanova, Miguel Ángel Sogorb, Jorge Estévez

**Affiliations:** Instituto de Bioingeniería, Universidad Miguel Hernández de Elche, Alicante, Spain

**Keywords:** Acetylcholinesterase, Assay, End-point, Fixed time, Sodium dodecyl sulphate

## Abstract

Many cholinesterase assays are performed to study the inhibition of cholinesterase (ChE) activity. Frequently a large number of samples are processed and Ellman's method [1] is the most commonly used [2,3]. Activity is estimated from the increment in absorbance between two reaction times when the reaction is not stopped. Bellino et al. [4] described a method based on Ellman's method whereby the reaction was stopped with SDS and then the absorbance was measured. In these methods, the chromogen reagent 5,5′dithiobis nitro benzoic acid (DTNB) is added with the substrate and colour is monitored. Some authors pointed that the chromogen can alter cholinesterase activity [5].

• A modification of Bellino's method is proposed for acetylcholine-hydrolyzing activity determinations that is based on stopping the reaction after a fixed substrate reaction time using a mixture of detergent SDS and DTNB.
• The method may be adapted to the user needs by modifying the enzyme concentration and applied for simultaneously testing many samples in parallel; i.e. for complex experiments of kinetics assays with organophosphate inhibitors in different tissues.

## Highlights

• An end-point method for cholinesterase determinations is performed.
• This method is based on stopping the reaction after a fixed reaction time.
• A large number of samples can be processed for complex kinetic assays.
• This assay can also be applied for manual, or with, automated workstations.
• This procedure allows to avoid undesired reactions by DTNB and TNB.

## Materials

### Chemicals

Sodium dodecyl sulphate (SDS; purity 99%) was obtained from Panreac Química S.L.U. (Barcelona, Spain). Triton X-100 was obtained from Sigma-Aldrich Quimica SL (Madrid, Spain). Ellman's reagent, 5,5′-dithio-bis-2-nitrobenzoate (DTNB, purity 99%) was obtained from Sigma–Aldrich Quimica SL (Madrid, Spain). Acetylthiocholine iodide (purity ≥ 98) was obtained from Sigma-Aldridge Quimica SL (Madrid, Spain). Phenylmethylsulfonyl fluoride (PMSF) was purchased from Sigma (Madrid, Spain), diethyl p-nitrophenylphosphate (paraoxon, purity >99%) was acquired from Sigma (Madrid, Spain). Human butyrylcholinesterase (hButChE) was supplied by Palmer W. Taylor and Zoran Radić (Skaggs School of Pharmacy and Pharmaceutical Sciences, University of California, San Diego, USA). Bovine serum albumin (BSA, purity 96%) was acquired from Sigma (Madrid, Spain). All the other reagents were obtained from Merck SL (Madrid, Spain) and were of analytical grade.

### Solutions

The “phosphate buffer” mentioned throughout the paper contained 0.1 M phosphate, pH 7.4, 1 mM EDTA.

The “phosphate buffer/6 mM DTNB” mentioned throughout the paper contained 0.1 M phosphate, pH 7.4, and 6 mM DTNB.

The “phosphate buffer/1% BSA” mentioned throughout the paper contained 0.1 M phosphate, pH 7.4, and 1% BSA.

The solution used to stop the AChE reaction contained 2% SDS and 6 mM DTNB, was prepared in the phosphate buffer, and is cited as “2% SDS/6 mM DTNB solution”.

Acetylthiocholine iodide was dissolved in ultrapure water at the desired concentration.

Thiocholine was obtained from the chemical degradation of acetylthiocholine iodide. A solution of 15 mM acetylthiocholine, pH 10, was incubated at 37 °C for 5 h. The resulting thiocholine preparation was neutralised at pH 7.4 and was diluted with ultrapure water at the desired concentration before use.

### Hen tissue preparation and subcellular fractioning

Hen tissues were obtained from a commercial slaughter house immediately after sacrifice. Brains were removed and stored in cold (0–5 °C). Tris buffer (50 mM Tris–HCl buffer at pH 8.0 containing 1 mM EDTA) until use (before 1 h). Brains were homogenised in a Polytron homogeniser (Kinematica GmbH, Germany) using a PTA 10S head at 70% power (3 × 3 s) in Tris buffer at a concentration of 200 mg fresh tissue/mL.

The homogenised tissue was centrifuged at 1000 × *g* for 10 min at 4 °C to yield a precipitate containing fibres and nuclei. The supernatant was then centrifuged at 100,000 × *g* for 60 min to precipitate mitochondrial and microsomal fractions. The pellet (containing fibres and nuclei) was resuspended with Tris–Triton buffer (50 mM Tris-HCl buffer at pH 8.0 containing 1 mM EDTA and 1% Triton X-100).

The supernatant (soluble fraction) and the resuspended pellet (membrane fraction) were kept in liquid nitrogen until use. Samples were thawed at room temperature before use. This concentrated enzyme preparation is cited thorough the paper as the “soluble enzyme preparation” or “membrane enzyme preparation” and was diluted with phosphate buffer at the desired concentration expressed as μL preparation/mL solution.

## Detailed method

In the following described procedure, each step was performed in all the test tubes before starting the next step. In this way, a large number of samples and blanks were simultaneously tested in parallel.

1. A 20-μL volume containing phosphate buffer (for blanks), or another reagent, was added to 1 mL microtubes. This volume may contain inhibitors or other factors that need to be tested.
2. Then 200 μL of the diluted membrane or soluble enzyme preparation (phosphate buffer in blanks) were added.
3. The mixture was incubated at 37 °C for the desired (preincubation) time. This preincubation time can be shortened substantially if inhibitors or other factors are not tested.
4. After this time, 200 μL of substrate acetylthiocholine in water was added for a final concentration of between 1 and 14.3 mM in 420 μL of the reaction volume.
5. The mixture was incubated at 37 °C for 10 min to run the enzymatic reaction.
6. The reaction was stopped by adding 200 μL of 2% SDS/6 mM DTNB solution.
7. Then 200 μL of phosphate buffer (diluted enzyme preparation in blanks) was added. The final assay volume was 820 μL.
8. After mixing and waiting at least 5 min, a 300-μL volume from each microtube was transferred to a 96-well microplate, and absorbance was read at 410 nm.

An automated Work Station (Beckman Biomek 2000) was employed, but the process can also be performed manually. By reducing all the volumes proportionally to ¼, for a final volume of 205 μL, the full process can be performed directly in a thermostat 96-well microplate.

The data recorded by the microplate reader were processed and graphic adjustments were made with the Sigma Plot software (Systat Software Inc, Chicago, USA) for Windows.

**Fig. 1:**
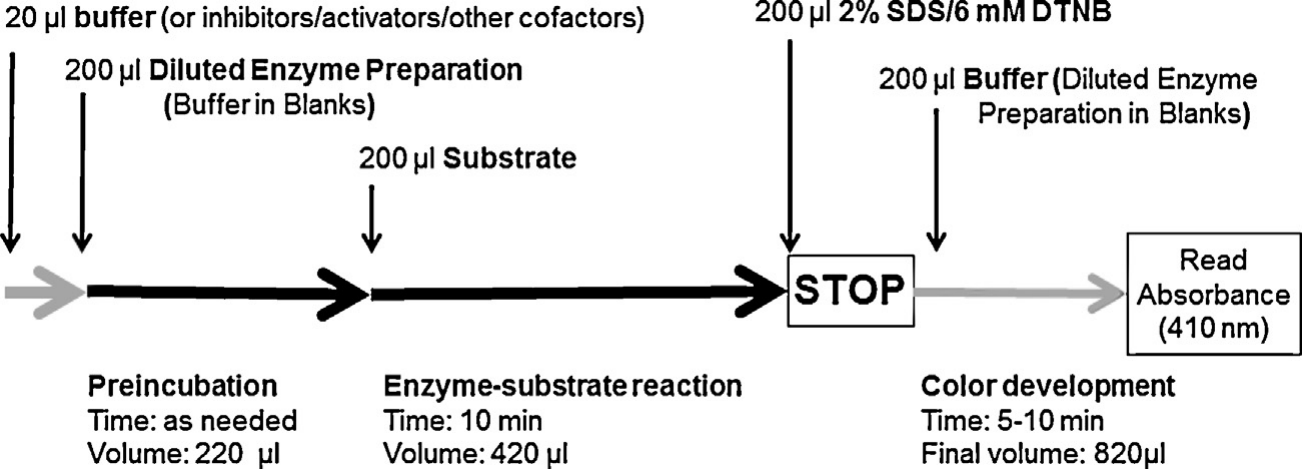
Method scheme. The whole procedure was performed at 37 °C.

Fig. 1 shows the timing of the procedure, while Table 1 provides a schematic summary of the assay protocol.

## Linearity of the colorimetric measure with a thiocholine concentration

A 220-μL volume of phosphate buffer and 200 μL of 2% SDS/6 mM DTNB were added to 200 μL of the indicated thiocholine solution. Absorbance was read at 410 nm.

**Fig. 2:**
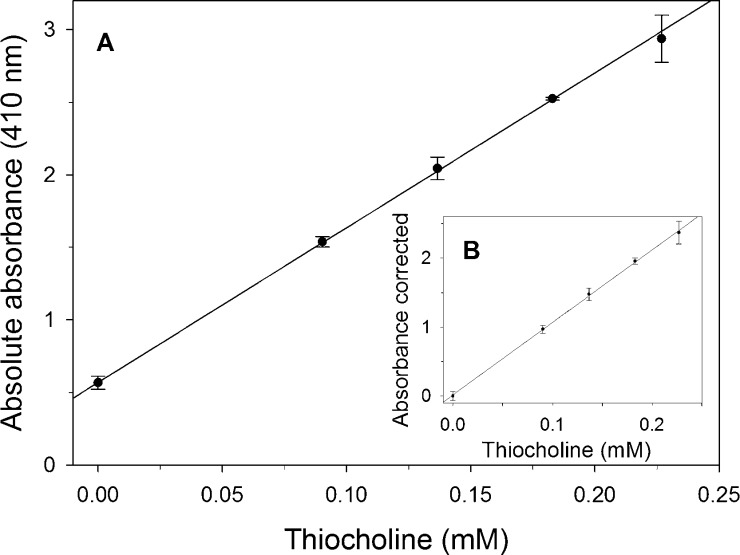
Thiocholine calibration curve. A 220-μL volume of thiocholine was incubated at 37 °C for 10 min with 200 μL of phosphate buffer. Then 200 μL of 2% SDS/6 mM DTNB solution was added. After that 200 μL of phosphate buffer/BSA was added. The thiocholine concentration in the assay volume (820 μL) was 0, 0.09, 0.14, 0.18 and 0.23 mM. Absorbance was measured at 410 nm. Panel A shows absolute absorbance (*n* = 3) and standard deviation. The linear regression parameters for the linear range were *y*_0_ = 0.569, *m* = 10.668 and *R*^2^ = 0.9951. Panel B shows the difference of absorbance corrected with the blanks (a solution without thiocholine containing the same 2% SDS/6 mM DTNB solution). The linear regression parameters were *y*_0_ = 0.016, *m* = 10.514 and *R*^2^ = 0.9994.

Fig. 2 shows that the absorbance at 410 nm was directly proportional to the thiocholine concentration (up to around 0.93 mM).

## Stability of absorbance

**Fig. 3:**
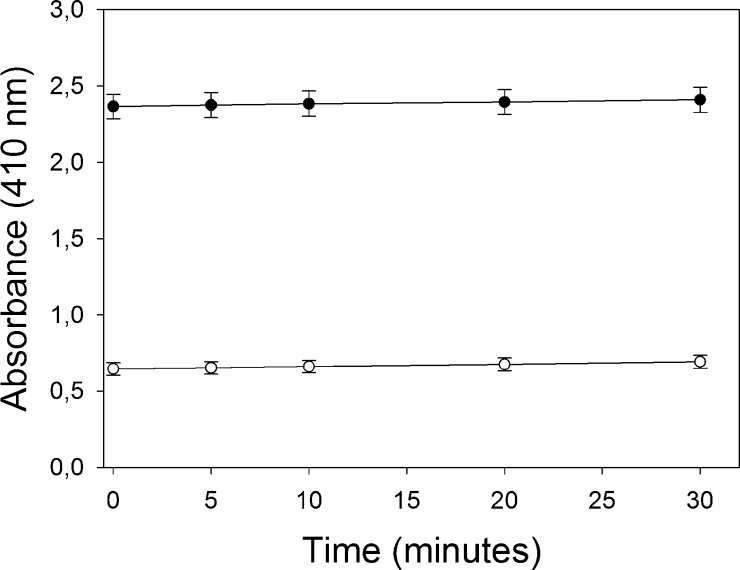
Stability of absorbance after stopping the enzyme-substrate reaction with soluble preparation. The absorbance (*n* = 3) of the enzyme-substrate reaction was measured. Black circles represent the absorbance of samples (50 μL soluble preparation/mL) according to the procedure described in Detailed Method. White circles represent the absorbance of the blanks. All the points represent the main value of three replicates and standard deviation is also represented.

**Fig. 4:**
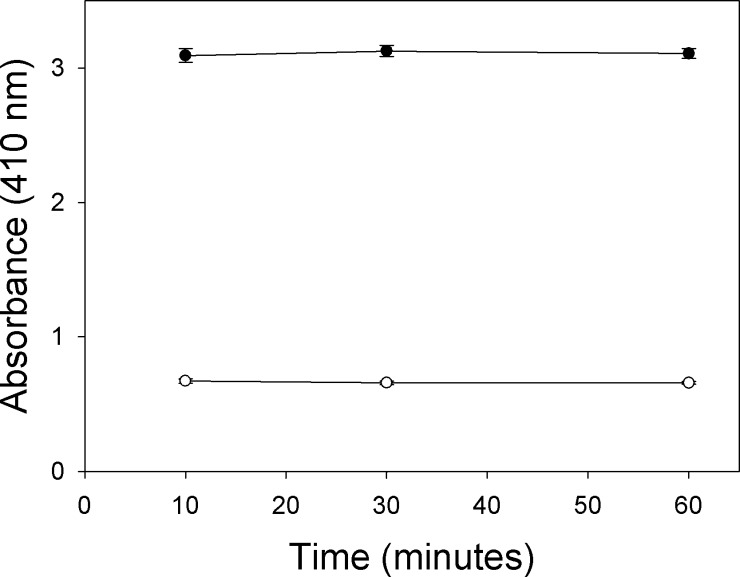
Stability of absorbance after stopping the enzyme-substrate reaction with membrane preparation. The absorbance (*n* = 3) of the enzyme-substrate reaction was measured. Black circles represent the absorbance of samples (50 μL membrane preparation/mL) according to the procedure described in Detailed Method. White circles represent the absorbance of the blanks. All the points represent the main value of three replicates and standard deviation is also represented.

Figs. 3 and 4 illustrate the stability of absorbance versus time in soluble or membrane preparations once the enzymatic reaction was stopped by adding the 2% SDS/6 mM DTNB solution according to the procedure described in Detailed Method. Blanks of acetylthiocholine chemical hydrolysis in which the diluted enzyme preparations was added after adding the 2% SDS/6 mM DTNB solution were also tested. If the absorbances of the diluted enzyme preparations and the blanks remained constant versus time, the reaction was considered to have been completely stopped. Therefore, the colorimetric measurement can be taken at any time to at least 30 min for soluble preparation and at least 60 min for membrane preparation after stopping the reaction without altering colour.

## Assay linearity with reaction time and amount of sample

A 20-μL volume of the phosphate buffer was incubated in 1 mL minitubes with 200 μL of different diluted enzyme preparations (21, 52.5 and 105 μL preparation/mL corresponding to 10, 25 and 50 μL soluble preparation/mL, respectively, in the 420-μL reaction volume) and with 200 μL of substrate (acetylthiocholine iodide at 30 mM corresponding to 14.29 mM in the 420 μL of reaction volume). After the reaction time (0, 2.5, 5, 10, 20 and 30 min) at 37 °C, 200 μL of 2% SDS/6 mM DTNB solution were added.

Samples with the same diluted enzyme preparation concentration, but with water with no substrate, were incubated at different reaction times. Spontaneous hydrolysis controls (samples without the diluted enzyme preparation) and controls of the colour produced by the 2% SDS/6 mM DTNB solution were included in the procedure.

**Fig. 5:**
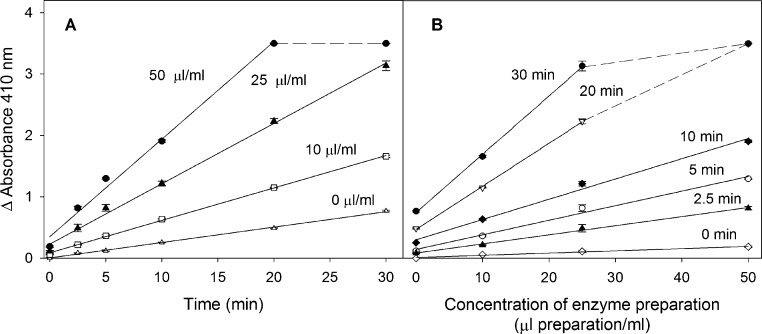
Assay linearity with time of reaction and tissue concentration. Panel A shows absorbance versus the reaction time for each different concentration of diluted enzyme preparation (μL preparation/mL). Panel B versus the concentration of diluted enzyme preparation for each reaction time. The points represent the main value of three replicates and standard deviation. The substrate concentration was 14.29 mM in the 420 μL of reaction volume.

Fig. 5 shows the linear dependence of activity versus reaction time for the different diluted soluble enzyme preparations (Fig. 5A), and versus the enzyme concentration for the various times (Fig. 5B). The response with the concentration of the diluted enzyme preparation was linear and time until absorbance was reached up to about 3.5 since the reaction was limited by the stoichiometry of the chromogenic reagent.

Slight absorbance was observed at 0 min of the reaction when the enzyme concentration increased. This was interpreted as being due to the reaction between DTNB and the thiol groups of the proteins in the diluted soluble enzyme preparation. Increased absorbance was also observed for the 0 μL preparation/mL due to some spontaneous hydrolysis of the substrate.

## Reproducibility assay

The proposed acetylcholine-hydrolysing activity assay described in Detailed Method was used to determine the activities of 12.5, 25, and 50 μL soluble preparation/mL with a substrate concentration of 14.28 mM acetylthiocholine in the reaction volume to study the variability of the intra-experiments values (Table 2). Variability was less than 0.8% for the highest tissue concentration and up to 4% for the lowest one.

Three independent experiments were performed on different days with a diluted soluble enzyme preparation of 25 μL soluble preparation/mL in the reaction volume and with a substrate concentration of 1 mM acetylthiocholine in the reaction volume to study the variability of the inter-experiments (*inter-die*) values (Table 3). The variability of each independent experiment was between 1.6 and 5.0%, whereas the variability of the averages among the three experiments was 6.45%. When considering all the data globally (*n* = 24), variability was 4.19%.

## Comparison between the usual kinetic Ellman's method and the proposed method

Kinetic Ellmans's method: 100 μL of 0.2 nM hButChE in phosphate buffer/1% BSA were mixed with 100 μL of phosphate buffer/6 mM DTNB and 20 μL of ultrapure water. After that 200 μL of 2.1 mM acetylthiocholine in water were added and incubated at 25 °C. Absorbance at 410 nm was read every 2 min since 3 min of starting the reaction until 17 min. The absorbance versus reaction time was lineal. The increase of absorbance between 5 and 15 min reaction time was 1.063 ± 0.047. A calibrate curve was performed in the same conditions. The lineal regression parameters were *y*_0_ = 0.004, *m* = 11.693 and *R*^2^ = 0.9896 from which the estimated specific activity was to 0.37 ± 0.02 mmol thiocholine/nmol hButChE/min.

Proposed fixed time endpoint method: 100 μL of 0.2 nM hButChE in phosphate buffer/1% BSA were mixed with 100 μL of phosphate buffer and 20 μL of ultrapure water for samples, or 100 μL phosphate buffer/1% BSA were mixed with 100 μL of phosphate buffer without EDTA and 20 μL of ultrapure water for blanks. After that 200 μL of 2.1 mM acetylthiocholine in water were added. The reaction was at 25 °C. After 10 min, the reaction was stopped adding 200 μL 2% SDS/6 mM DTNB and then, 100 μL phosphate buffer/1% BSA and 100 μL of phosphate buffer without EDTA were added to samples, and 100 μL of 0.2 nM hButChE in phosphate buffer/BSA and 100 μL of phosphate buffer without EDTA were added to blanks. The absorbance was read at 410 nm. The corrected absorbance (difference with blank) was 0.862 ± 0.010. The activity was estimated to be 0.66 ± 0.03 mmol thiocholine/nmol hButChE/min.

The activity obtained by the proposed fixed time endpoint method is higher than the activity obtained through the usual kinetic Ellman's method. This difference of activities can be explained because DTNB interacts with the protein [5] in the usual kinetic Ellman's method because DTNB is in the medium during the enzymatic reaction.

## Figures and Tables

**Table 1:** End-point protocol for measuring acetylcholinesterase. Each step is performed with each sample/tube to be tested before starting the next step. This strategy allows testing many samples in parallel.

Step	Action
(1)	20 μL of buffer (or inhibitors/activators/cofactors)^a^
(2)	200 μL of diluted Enzyme preparation (buffer in Blanks) and mixing^b^
**(3)**	*(Total preincubation volume: 220* *μL)***Preincubation time (as needed)**
(4)	200 μL of substrate and mixing^c^
**(5)**	*(Total enzyme reaction volume: 420* *μL)***Enzyme reaction time**: 10 min at 37 °C
**(6)**	**Stop enzyme reaction** by adding 200 *μL* of 2% SDS/6 mM DTNB and mixing
(7)	200 μL of buffer (diluted Enzyme preparation in blanks) and mixing^d^
(8)	*(Total final volume for the colorimetric measure: 820* *μL)*^e^ Wait 5–10 min
**(9)**	**Read absorbance at 410** **nm** Estimate of corrected absorbance (Samples–Blanks) Calculations: e.g.,: units of activity (nmol/min), percent over controls, others

a The 20 μL volume is reserved for adding inhibitors, activators, cofactors or other reagents.

b Blanks are intended to include colour due to: (i) substrate spontaneous hydrolysis; (ii) the DTNB reaction with the protein thiol groups in the enzyme preparation; (iii)the background absorbance of DTNB, enzyme preparation, plastic ware, others.

c Acetylthiocoline to obtain the desired concentration with the 1–15 mM range in the enzyme reaction volume (420 μL).

d Adding this final volume in blanks and samples can be avoided if it has been previously demonstrated that the colour increase caused by the enzyme preparation is negligible.

e Final volumes may be reduced by maintaining the same proportions of all reagents. For example: 5 μL buffer/inhibitors + 50 μL enzyme + 50 μL substrate + 50 μL SDS/DTNB + 50 μL buffer for a final volume of 205 μL to perform the full process in a 96-wel microplate.

**Table 2:** Intra-assay reproducibility. The experiment was performed according to the assay described in Detailed Method. The substrate concentration was 14.28 mM acetylthiocholine in the reaction volume and the reaction time was 10 min. The activity was estimated according to the linear regression parameters obtained in the thiocholine calibration curve (Fig. 2).

Tissue concentration	Activity ± SD (nmol/min)	% SD	Activity in the preparation (nmol/min/μL preparation)
12.5 μL preparation/mL (*n* = 8)	3.07 ± 0.11	3.7	0.585 ± 0.022
25 μL preparation/mL (*n* = 8)	5.42 ± 0.11	2.06	0.516 ± 0.011
50 μL preparation/mL (*n* = 8)	12.47 ± 0.10	0.76	0.594 ± 0.005

**Table 3:** *Inter-die* assay reproducibility. Three independents experiments were performed according to the assay described in detailed method. Each experiment was assayed on different days. The substrate concentration was 1 mM acetylthiocholine in the reaction volume and the reaction time was 10 min. The activity was estimated according to the linear regression parameters obtained in the thiocholine calibration curve (Fig. 2).

	Activity ± SD (nmol/min)	% SD	Activity in the preparation (nmol/min/μL preparation)
Experiment 1 (*n* = 8)	7.54 ± 0.38	5.02	0.718 ± 0.036
Experiment 2 (*n* = 8)	7.35 ± 0.12	1.62	0.700 ± 0.011
Experiment 3 (*n* = 8)	6.99 ± 0.25	3.63	0.666 ± 0.024
Average of the experiment (*n* = 3)	7.29 ± 0.47	6.45	0.695 ± 0.045
Global (*n* = 24)	7.29 ± 0.31	4.19	0.695 ± 0.029
